# A controlled trial of Partners in Dementia Care: veteran outcomes after six and twelve months

**DOI:** 10.1186/alzrt242

**Published:** 2014-02-28

**Authors:** David M Bass, Katherine S Judge, A Lynn Snow, Nancy L Wilson, Robert O Morgan, Katie Maslow, Ronda Randazzo, Jennifer A Moye, Germaine L Odenheimer, Elizabeth Archambault, Richard Elbein, Paul Pirraglia, Thomas A Teasdale, Catherine A McCarthy, Wendy J Looman, Mark E Kunik

**Affiliations:** 1 Benjamin Rose Institute on Aging, 11890 Fairhill Road, Cleveland, OH 44120, USA; 2 Department of Psychology, Cleveland State University, 2121 Euclid Ave., Cleveland, OH 44115, USA; 3 Center for Mental Health and Aging and Department of Psychology, University of Alabama, Box 870315, Tuscaloosa, AL 35487, USA; 4 Tuscaloosa Veterans Affairs Medical Center, 3701 Loop Rd., Tuscaloosa, AL 35404, USA; 5 Houston Veterans Affairs Health Services Research & Development Center for Innovations in Quality, Effectiveness and Safety, Michael E. DeBakey Veterans Affairs Medical Center, (MEDVAMC 152), 2002 Holcombe Blvd., Houston, TX 77030, USA; 6 Baylor College of Medicine, One Baylor Plaza, Houston, TX 77030, USA; 7 The University of Texas School of Public Health, 1200 Herman Pressler, Rm. E-343, Houston, TX 77030, USA; 8 Institute of Medicine, 2101 Constitution Ave. NW, Washington, DC 20418, USA; 9 Alzheimer’s Association of Massachusetts/New Hampshire, 480 Pleasant St., Watertown, MA 02472, USA; 10 Geriatric Mental Health, Veterans Affairs Boston Healthcare System, Brockton Division, 940 Belmont St., 35C, Brockton, MA 02301, USA; 11 Department of Psychiatry, Harvard Medical School, 2 West – Rm. 305, 401 Park Drive, Brockton, MA 02215, USA; 12 Oklahoma City Veterans Affairs Medical Center, 921 NE 13th St., Oklahoma City, OK 73106, USA; 13 Donald W. Reynolds Department of Geriatric Medicine, College of Medicine, University of Oklahoma Health Sciences Center, P.O. Box 26901, ORB 1200, Oklahoma City, OK 73117, USA; 14 Veterans Affairs Boston Health Care System, West Roxbury Division, 1400 VFWParkway, West Roxbury, MA 02132, USA; 15 Houston & Southeast Texas Chapter, Alzheimer’s Association, 2242 W. Holcombe Blvd., Houston, TX 77030, USA; 16 Providence Veterans Affairs Medical Center, 830 Chalkstone Ave., Providence, RI 02908, USA; 17 Brown University, 1 Prospect St., Providence, RI 02912, USA; 18 South Central Veterans Affairs Mental Illness Research, Education and Clinical Center, 2002 Holcombe Blvd, Houston, TX 77030, USA; 19 Alzheimer’s Association of Massachusetts/New Hampshire, 5 Bedford Farms Drive, Ste. 201, Bedford, NH 03110, USA

## Abstract

**Introduction:**

“Partners in Dementia Care” (PDC) tested the effectiveness of a care-coordination program integrating healthcare and community services and supporting veterans with dementia and their caregivers. Delivered via partnerships between Veterans Affairs medical centers and Alzheimer’s Association chapters, PDC targeted both patients and caregivers, distinguishing it from many non-pharmacological interventions. Hypotheses posited PDC would improve five veteran self-reported outcomes: 1) unmet need, 2) embarrassment about memory problems, 3) isolation, 4) relationship strain and 5) depression. Greater impact was expected for more impaired veterans. A unique feature was self-reported research data collected from veterans with dementia.

**Methods and Findings:**

Five matched communities were study sites. Two randomly selected sites received PDC for 12 months; comparison sites received usual care. Three structured telephone interviews were completed every 6 months with veterans who could participate.

**Results:**

Of 508 consenting veterans, 333 (65.6%) completed baseline interviews. Among those who completed baseline interviews, 263 (79.0%) completed 6-month follow-ups and 194 (58.3%) completed 12-month follow-ups. Regression analyses showed PDC veterans had significantly less adverse outcomes than those receiving usual care, particularly for more impaired veterans after 6 months, including reduced relationship strain (B = −0.09; p = 0.05), depression (B = −0.10; p = 0.03), and unmet need (B = −0.28; p = 0.02; and B = −0.52; p = 0.08). PDC veterans also had less embarrassment about memory problems (B = −0.24; p = 0.08). At 12 months, more impaired veterans had further reductions in unmet need (B = −0.96; p < 0.01) and embarrassment (B = −0.05; p = 0.02). Limitations included use of matched comparison sites rather than within-site randomization and lack of consideration for variation within the PDC group in amounts and types of assistance provided.

**Conclusions:**

Partnerships between community and health organizations have the potential to meet the dementia-related needs and improve the psychosocial functioning of persons with dementia.

**Trial Registry:**

NCT00291161

## Introduction

More than 300,000 veterans with dementia receive care from the Department of Veterans Affairs (VA), the largest healthcare system in the United States [1]. To address the complex and diverse care issues associated with this disorder, the VA is working to develop a comprehensive system of support services for veterans with dementia and their informal caregivers [2,3]. The current investigation, ‘Partners in Dementia Care’ (PDC), was highlighted as one of the VA’s dementia initiatives that tested the effectiveness of a care-coordination program designed to integrate health care and community services through structured coaching and support [4]. Coordination of health and community services is a priority area of the recently enacted National Plan to Address Alzheimer’s Disease [5].

PDC is a version of the evidence-based program, ‘BRI Care Consultation,’ which was developed by a research team led by the Benjamin Rose Institute on Aging to assist older adults and caregivers dealing with chronic health conditions [6-8]. The Benjamin Rose Institute on Aging holds the copyright to BRI Care Consultation and currently licenses and trains organizations to deliver the program. Since completion of PDC, more than two dozen diverse organizations have been licensed to deliver BRI Care Consultation, including healthcare organizations, Alzheimer’s Association chapters, family counseling agencies and Area Agencies on Aging.

A key feature of PDC is its basis in a formal partnership between a healthcare organization (for example, VA medical centers) and a community service organization (for example, Alzheimer’s Association chapters). Bringing healthcare and community providers together in this partnership was designed to facilitate more holistic care that: 1) improved fragmentation and lack of coordination between medical care and community services [9,10]; 2) raised awareness of healthcare providers about nonmedical needs of patients and caregivers [11,12]; 3) increased information and educational resources on dementia, its care and illness-related strain [13-15]; 4) reduced difficulties accessing and monitoring services [9]; and 5) improved management of dementia with coexisting medical conditions [16,17].

PDC gives equal attention to veterans with dementia and their primary informal caregivers (that is, family member or friend). Veterans are active participants in PDC whenever possible, despite their dementia. Involving persons with dementia, as well as their caregivers, distinguishes PDC from many of the more than 40 evidence-based, non-pharmacological interventions that focus exclusively on caregivers [18]. Moreover, PDC is one of the few evidence-based programs that assessed impact by examining self-reported outcomes derived directly from persons with dementia. More commonly, outcomes come from proxy reports of caregivers that may not accurately depict the person’s subjective views [19,20].

This research was a controlled trial that tested the effectiveness of PDC by examining the impact on self-reported outcomes by veterans with dementia. The study was approved by the institutional review boards of the Providence VA Medical Center, VA Boston Healthcare System, University of Oklahoma Health Sciences Center and Baylor College of Medicine.

The Stress Process Model, which has been widely used in research on stress and coping for more than 30 years and recently was adapted for individuals with dementia (9), guided the two study hypotheses [21-23]. Key multidimensional domains in the Stress Process Model are: 1) objective and subjective primary stressors, 2) role and intrapsychic strain, 3) internal and external support resources, and 4) well-being [21]. Primary objective and subjective stressors are the amount and type of impairments experienced by the individual. Secondary role and intrapsychic strains are negative consequences of primary stressors for fulfilling social roles (for example, work and family) and for internal psyche (for example, feelings of embarrassment and isolation). Internal and external support resources, such as social support and services, can directly or indirectly reduce negative consequences of stressors. Well-being is the net result of the other three domains and includes global constructs such as depression and anxiety.

This study’s two hypotheses are based on conceptualizing PDC as an external support resource (domain 3) that may directly or indirectly reduce negative consequences of primary stressors, which are veterans’ impairments. The first hypothesis focuses on direct benefits and posits that veterans who receive PDC will have decreases in five adverse veteran-reported outcomes that represent four types of role and intra-psychic strains (domain 2): 1) unmet need for help or information, 2) embarrassment about memory problems, 3) isolation from others and 4) relationship strain between veterans and their primary informal caregivers), and 5) one component of well-being (domain 5) (symptoms of depression). The second hypothesis focuses on indirect effects of PDC on the same five outcomes, with the potential benefits being conditional or dependent on severity of, or difficulty with, veterans’ impairments. The conditional-effects hypothesis posits that PDC will have greater benefits for veterans with more severe cognitive impairment or more dependencies in personal care. This hypothesis is based on the Stress Process Model’s assumption that negative effects of a chronic stressor, such as dementia, are not uniform across all individuals but depend upon the severity of symptoms and perceptions of difficulties associated with the stressor [24,25].

## Methods

### Design

There were five study sites: Boston, MA; Houston, TX; Providence, RI; Oklahoma City, OK; and Beaumont, TX, with all sites located in one of two selected Veterans Integrated Service Networks (VISNs). VISNs provide a unifying administrative structure for all VA services within a given geographic region. Study sites were matched by VISN to assure uniformity in this overarching administrative structure. One of the two selected VISNs (that is, VISN 16, which includes Houston, Oklahoma City and Beaumont) was chosen because it was the location of the study’s VA principal investigator. The other selected VISN (that is, VISN 1, which includes Boston and Providence) had a similar array of VA services as VISN 16.

Within each of the two selected VISNs, VA study sites were matched to be similar in: size, services offered (both inpatient and outpatient), academic affiliations, research missions and medical-residency training programs. Alzheimer’s Associations chapters within selected VISNs were similar in size, with comparable core programs and services. After selecting matched sites within each VISN, one was randomly selected to deliver PDC and the other was deemed the comparison site that would deliver usual care (UC). Specifically, in one VISN, Boston was randomly selected to deliver PDC and Providence was selected as its matched UC site. In the other VISN, Houston was randomly selected to deliver PDC and Oklahoma City was selected as its matched UC site. Beaumont was paired with Oklahoma City as part of the same UC site to assure a sufficiently sized comparison sample. Matched PDC and UC sites, rather than within-site randomization, were used to allow PDC implementation throughout partnering organizations, without concerns about diffusion of PDC to the UC group.

Veterans and caregivers at PDC sites received the care-coordination program and an initial, basic packet of educational materials on dementia; UC-site veterans and caregivers received the same basic educational materials and usual care from the VA and chapters. Aside from PDC care coordination, there were no restrictions on services or care that could be obtained by the PDC or UC groups from the VA, chapters or other organizations.

### Sample

Eligibility requirements for veterans included receiving primary healthcare from the VA, residing outside a residential care facility at the time of enrollment, living within a partnering chapter’s service area, being 60+ years of age and having at least one of the following dementia diagnostic codes from the International Classification of Diseases, Ninth Revision recorded in the VA medical record: 290.41–43, 291.2, 292.82, 294.1, 294.8 and 331.0. There was no enrollment restriction based on type or severity of veterans’ impairments, or availability of a family caregiver. VA primary care physicians confirmed veterans’ diagnoses and eligibility prior to sample selection.

The sample was recruited from 18 January 2007 to 22 July 22 2009. Research interviews occurred from 21 March 2007 to 15 September 2010. Veterans’ ability to complete research interviews that provided self-reported outcomes was determined by a telephone screening, using the short Blessed Orientation-Memory-Concentration Test [26]. The Blessed Test comes from a longer instrument and was one of the tools recommended in a dementia diagnostic protocol published by the Agency for Health Care Research and Policy [27]. It includes six items that assess time orientation, ability to count backwards from 20, ability to say months in reverse order and ability to repeat a simple phrase that includes a name and address. The Blessed Test was successfully used as a brief instrument for measuring cognitive status over the telephone in prior studies of BRI Care Consultation [6]. Because of its ease of use and the lack of other tested methods for deciding whether a person with dementia could complete a research interview focused on self-reported outcomes, the Blessed Test was adapted as a screening device for determining whether veteran interviews were attempted. In this adaptation, three criteria were used. First, veterans had to be able to give answers to all six questions over the telephone, even if there were errors in the answers. Second, veterans had to be able to repeat accurately at least two parts of a three-part phrase immediately after the telephone screener stated it to them. Third, veterans either had to count backwards accurately from 20 to 15 or accurately name at least three consecutive months in reverse.

Sample-size estimates were based on a one-tailed significance test, a modest effect size (.12), .80 power, an alpha of .05, 10 independent variables in a regression equation and an estimated squared multiple correlation of .55. A minimum sample size of 247 was determined based on these assumptions.

### PDC model intervention

Two half-time care coordinators, with part-time administrative assistant support, delivered PDC at each intervention site. One care coordinator worked in the local VA medical center (healthcare organization) and the other worked in the partnering Alzheimer’s Association chapter (community service organization). Although from different organizations, the two care coordinators worked as a team, with one shared electronic Care Coordination Information System (CCIS) and regularly scheduled planning and case-conference meetings. Care coordinators had bachelor’s or master’s degrees in social work, nursing or other helping professions.

The care coordinator from the VA medical centers had primary responsibility for assisting veterans with medical-related concerns (for example, medications, accessing medical services, disease management) while the care coordinator from the Alzheimer’s Association chapter had primary responsibility for assisting caregivers with nonmedical concerns (for example, care-related strain, accessing family support and information services). The VA care coordinator also focused on helping families access VA services and benefits, whereas the Alzheimer’s Association care coordinator focused on helping families use community services, including those offered by the Alzheimer’s Association. This division of labor between care coordinators capitalized on the complementary strengths of each partner organization and represented a bridge between health care and community services.

Training for care coordinators consisted of a 1.5-day initial session on the PDC philosophy, service-delivery protocol and the CCIS that guides service delivery. Additionally, one- to two-hour biweekly refresher trainings were completed throughout the study period. These sessions focused on case reviews to monitor fidelity to the intervention protocol, strategies for working with a partner organization, using the CCIS and handling difficult cases. Continuing education also was provided on special topics, such as differences among illnesses that cause dementia, helping families respond to emergencies and respite for caregivers.

PDC is a coaching model driven by consumer choice, with care coordinators helping find solutions to concerns that are the priorities of veterans and caregivers. PDC followed a set, standardized protocol that required a minimum of at least one contact between care coordinators and consumers per month; more-frequent contacts occurred as needed. The protocol required care coordinators to discuss with veterans and/or caregivers a broad range of medical and nonmedical concerns, although the specific content was customized to consumers’ preferences and needs.

PDC is a low-cost service delivered by telephone, mail and e-mail, with in-person contacts rarely needed. The two half-time care coordinators from the partnering organizations (one full-time equivalent (FTE)) maintained caseloads of 75 to 125 families. All expenses to deliver PDC (that is, salaries, benefits, equipment, supplies, training, software, licensing, supervision, administrative overhead) can be recovered by charging a fee of $60 to $80 per month per family.

PDC gives equal attention to preferences and needs of veterans and caregivers. Veterans with dementia are engaged in the program whenever possible, despite their impairments. Veterans without caregivers are able to use PDC, so long as they can communicate by telephone. If veterans are too impaired to communicate by telephone, their caregivers can be the sole participant in the program.

PDC has three main components: 1) initial assessment, 2) action plan, and 3) ongoing monitoring and reassessment.

#### Initial assessment

The initial assessment is completed gradually during the first four weeks of enrollment. It is designed to be brief, with the action plan to address assessed concerns implemented simultaneously with or prior to completion of the entire initial assessment. The initial assessment covers a broad range of domains or potential problem areas: 23 for veterans (for example, coordinating and accessing VA services, medication management, getting and understanding the diagnosis) and 16 for caregivers (for example, finding and accessing community services, care-related strains and depression). The required initial assessment consists of a single-item trigger question for each domain; trigger questions can be formally asked or covered informally during conversations. More extensive detailed assessment questions are available for each domain as optional tools, if additional probing is necessary to clarify a problem.

#### Action plan

The action plan is the core of PDC. It comprises simple behavioral tasks called action steps that, if accomplished, move veterans and caregivers toward solutions to concerns they identified as important. Action steps should be easy to complete and include, for example, calling an organization to inquire about the availability of a service, reading an educational resource on a topic of concern or contacting another family member to ask whether he or she is willing to help with a caregiving task. With coaching and guidance from care coordinators, veterans and caregivers determine the content of action steps, who will complete the action steps and the projected dates of completion. New action steps are continuously added and build upon prior action steps. Multiple action steps, spread over a period of weeks or months, often are needed to find solutions to specific problems. As action steps are completed, veterans and caregivers move toward solutions and gain confidence in their self-management abilities. Copies of action plans are mailed to veterans and caregivers and summarized in the larger medical record.

On average, each veteran and his or her caregiver had more than seven action steps. The most common pertained to accessing and coordinating services and benefits available from the VA, Alzheimer’s Association or other community agencies. Specifically, 78% of veterans and caregivers had action steps related to accessing VA services or benefits, 59% to accessing Alzheimer’s Association services and 76% to accessing other community organizations. Other common action steps focused on improving care from the informal network (57%), managing symptoms (40%), improving communication with healthcare providers (33%) and home safety (29%).

#### Ongoing monitoring and reassessment

The hallmark of PDC is a long-term relationship to provide continuous support to veterans and caregivers. Ideally, care coordinators become knowledgeable and familiar experts who are trusted by families. On average, families had over 14 contacts with coordinators during the twelve-month study period, which focused on completing the required initial assessment and reassessments, adding new action steps and checking the disposition of pending action steps, and completing required routine checking.

Reassessments involved readministering trigger questions used in the initial assessment. They were required at least every six months. More frequent reassessments for selected domains are recommended for persistent or ongoing problems. Reassessment helps care coordinators and consumers gauge progress in finding solutions to problems.

Consistent with the design of PDC, the most contacts between care coordinators and veterans or caregivers were by telephone (80%) and regular mail and e-mail (16%), with a small number in person (4%). The number of contacts was evenly split between care coordinators from the VA and the Alzheimer’s Association, which reflected PDC’s team-based delivery model. Care coordinators initiated approximately 90% of contacts; veterans or caregivers initiated 10%. (For a more detailed description of PDC, see Judge *et al*. [28]).

### Data collection

The study period was twelve months, with three research telephone interviews attempted with participating veterans who were able to pass the baseline telephone screening with the adapted Blessed Test and three research interviews with veterans’ caregivers. Research interviews were administered by trained interviewers. Baseline interviews were conducted as soon as possible after written consents were received. To assess the impact of PDC on study outcomes, second and third research interviews were conducted at six and twelve months postbaseline.

### Outcomes measures

Five self-reported outcomes from veterans with dementia (that is, 1) unmet needs, 2) embarrassment about memory problems, 3) isolation, 4) relationship strain and 5) depression) were examined after six and twelve months. As described below, psychometric properties of these outcomes were tested with the current sample of veterans to reaffirm their reliability and structural validity, which had been established in previous studies for all but one outcome (citations provided below). Overall, psychometric analyses showed individual items comprising these outcomes had good internal reliability, based on Cronbach’s alpha, and good structural validity, based on high factor loadings on a single factor and low cross-loadings on factors representing other outcomes. Thus, psychometric analyses reinforced the feasibility of collecting information from persons with dementia, despite mild-to-moderate levels of cognitive impairment. However, a limitation of the outcomes was the use of simple ‘yes/no’ response choices for most questions comprising these measures to facilitate administration with cognitively impaired respondents [29].

#### Unmet needs

Developed for this study, this outcome was based on 24 dichotomous questions that were summed to measure veterans’ perceptions of unmet need across eight domains: 1) understanding dementia, 2) daily living tasks, 3) accessing VA and other services, 4) legal and financial issues, 5) organizing family care, 6) alternative living arrangements, 7) emotional support and 8) medications. This measure had good structural validity, with factor loadings from .83 to .62 on a single factor and good internal reliability (Cronbach’s alpha .93, .90 and .93 at baseline, six months and twelve months, respectively).

#### Embarrassment about memory problems

This previously published outcome [30] was the sum of three dichotomous items that asked whether veterans felt embarrassed about memory problems, uncomfortable telling others about memory problems and uncomfortable accepting help for memory problems (Cronbach’s alpha .75, .74 and .74 at baseline, six months and twelve months, respectively). Factor analysis confirmed the independence and structural validity, with loadings from .53 to .67.

#### Isolation

This previously published outcome [30] included four dichotomous items and asked veterans whether their health problems and need for assistance made them feel isolated from other people, less able to participate in group activities, less able to participate in church or religious activities, and less able to visit with family and friends (Cronbach’s alpha .77, .77 and .82 at baseline, six months and twelve months, respectively). Factor loadings for these items ranged from .39 to .84.

#### Relationship strain

This outcome was adapted from a published family caregiving measure [31] and was the sum of four dichotomous items focused on veterans’ perceptions of the quality of the relationship with their caregivers. Questions asked whether, because of their health problems and need for assistance, veterans felt that their caregiver tried to manipulate them, felt that the relationship with the caregiver was strained, felt resentful toward the caregiver or felt angry toward the caregiver (Cronbach’s alpha .78, .77 and .84 at baseline, six months and twelve months, respectively). Factor loadings for these items ranged from .42 to .85.

#### Depression

Veteran depression was measured by the 11-item Center for Epidemiologic Studies Depression Scale, which had good reliability at all three data-collection waves (Cronbach’s alpha of .76, .79 and .78 at baseline, six months and twelve months, respectively) [32].

### Impairment measures

Two multi-item scales representing impairment were used to test the conditional-effects hypothesis: 1) cognitive impairment was measured by the Blessed Test [26]; and 2) number of personal-care dependencies with bathing, dressing, grooming, toileting, eating and mobility inside a house (Cronbach’s alpha of .73, .82 and .81 at baseline, six months and twelve months, respectively) [33]. For hypothesis testing, the analyses used the veteran’s full score on the Blessed Test, in contrast to the previously described adaptation to determine whether a research interview was attempted. Follow-up interviews at six and twelve months were attempted with all veterans who passed the initial screening and completed baseline interviews.

### Covariates

A wide range of baseline measures was used to test for initial differences between PDC and UC groups that may have resulted from using matched sites rather than randomization.

### Analytic strategy

Multiple regression equations tested whether veterans in the PDC and UC groups differed across the five outcomes: 1) unmet need, 2) embarrassment, 3) isolation, 4) relationship strain, and 5) depression. Two equations were estimated for each: one representing change from baseline to six months and one representing change from six to twelve months. In each equation, the effect of PDC on an outcome was represented by the regression coefficient for a dichotomous variable that distinguished between veterans in the PDC and UC groups. In addition, two product or interaction terms tested the conditional-effects hypothesis that posited the benefits of PDC would be greater for more impaired veterans (for example, those with more cognitive impairment and/or personal-care dependencies). Product terms were constructed by multiplying the dichotomous intervention variable by the two variables representing impairments. To facilitate interpretation, impairment measures were centered to have a mean of zero [34].

Regression coefficients for the PDC variable and product terms were of primary interest to this study. When there were no significant product terms and the regression coefficient for the PDC variable was significant, it meant the difference between the PDC and UC groups pertained to all veterans, regardless of level of impairment (that is, no conditional effects). When there was a significant product term, the regression coefficient for the PDC variable represented the difference between the PDC and UC groups among veterans with mean levels of cognitive impairment or personal-care dependencies. The regression coefficient for a significant product term represented the average difference in an outcome between PDC and UC groups among veterans with higher- and lower-than-average levels of impairment. (See Cohen and Cohen [35] or McClendon [34] for a detailed discussion of interpreting product terms in regression analysis).

Final regression equations included the dichotomous PDC variable and any significant product terms, measures of veterans’ cognitive impairment and personal-care dependencies, the prior-wave’s version of the dependent variable and four background characteristics to control for significant baseline differences between the PDC and UC groups. By including the prior-wave’s version of the dependent variable, equations focus on ‘changes’ in outcomes from baseline to six months or from six to twelve months. Based on the coding of the intervention variable (that is, 1 = PDC group; 0 = UC group), results consistent with hypotheses were indicated by significant regression coefficients with negative values. Analyses were run using IBM SPSS Statistics, Version 21 (Armonk, NY).

## Results

**Figure 1:**
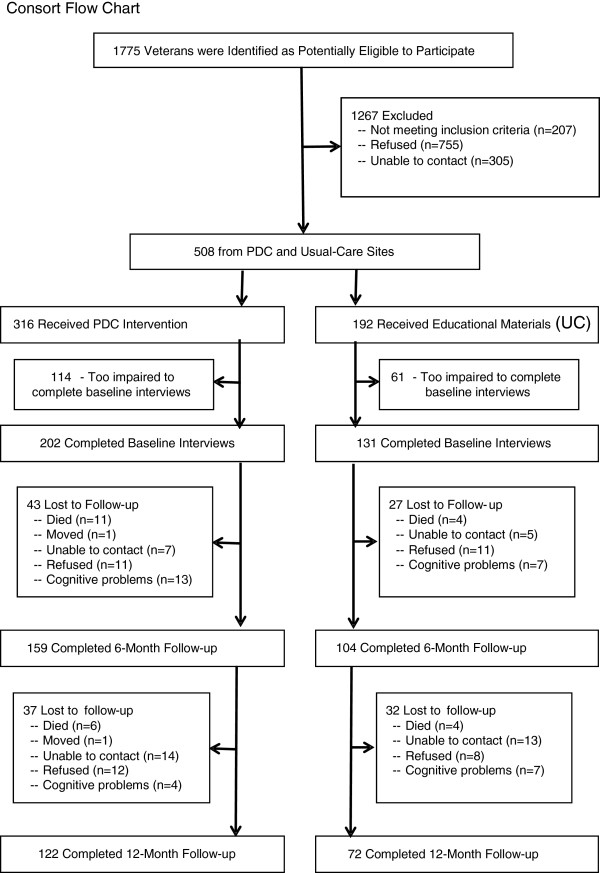
**CONSORT flow chart.** This diagram shows the flow of participants by group from identification to final 12-month assessment.

A total of 1,775 veterans were referred to the project by VA primary care physicians and mailed IRB-approved study invitations and consent forms. Signed consent forms were received for 508 veterans (28.6%). All but 22 consenting veterans had a family or friend caregiver who also participated in the study. Among nonparticipating veterans, 207 (11.7%) were ineligible (for example, died, were in a nursing home), 305 (17.2%) were not able to be reached by telephone after mailing the study invitations and 755 (42.5%) actively or passively declined. Figure 1 provides a Consort Flow Chart that describes the study sampling process.

Nearly all veterans were men (97.5%) and nearly all caregivers were women (94.9%). The majority of veterans (76.4%) completed at least high school, with 24.8% having a college degree; and 19.0% identified themselves as a member of a minority group. On average, veterans received their dementia diagnosis 2.03 years (SD = 1.97) prior to the study.

Of the 508 consenting veterans, 333 (65.6%) passed the telephone screening using the adapted Blessed Test and completed the baseline research interview. The second research interview, conducted six months post-baseline, measured outcomes after a shorter period of study participation and was completed by 263 of the 333 veterans (79.0%). The third research interview, conducted 12 months post-baseline, measured outcomes after a longer period of study participation and was completed by 194 of the 333 veterans (58.3%). Attrition analysis comparing veterans who completed baseline interviews but who did not complete six- and/or twelve-month follow-ups indicated that those who stopped participating were significantly more likely to be members of a minority group, more impaired in personal care at baseline, and more isolated from others at baseline. These differences suggest study results may not fully represent the experiences of veterans who are more vulnerable or disadvantaged in terms of health, social support and socioeconomic status.

Although this study demonstrates the feasibility of collecting data directly from persons with dementia, it is important to note that 175 (34.4%) veterans participating in PDC were too impaired to be interviewed at baseline and were not represented. Additionally, as described in Figure 1, a number of veterans who completed baseline interviews did not complete six- and twelve-month follow-ups due to their worsening symptoms or death. Thus, findings do not represent more severely impaired veterans.

Table 1 describes baseline characteristics of the sample. The average level of personal-care dependencies was 1.91 tasks on a scale from zero to six tasks, indicating many veterans were still independent in more than half of the personal-care tasks, such as bathing and dressing. Cognitive impairment, measured by the Blessed Test, had a mean of 11.24 and a standard deviation of 5.94, indicating considerable diversity in this characteristic. This variation is consistent with study-recruitment procedures that did not restrict participation based on stage of dementia. Scores on the Blessed Test can range from zero (no errors on test questions and low cognitive impairment) to 28 (all test questions in error and high cognitive impairment). In this sample, a score of 26 was the most errors by any veteran, with approximately 11% of the sample having 20 or more errors. Descriptive information for all other variables in the analysis is presented in Table 1.

**Table 1:** Descriptive statistics for baseline variables used in the analysis of veteran outcomes

	**Total (Number = 333)**		**PDC group (number = 202)**		**Usual care group (number = 131)**		
	**% or mean**	**St. dev.**	**% or mean**	**St. dev.**	**% or mean**	**St. dev.**	**t**
**Intervention group**	60.7%	–	–	–	–	–	
**Veteran outcomes**							
Unmet needs (0 to 24, low to high)	6.24	6.56	6.88	6.90	5.23	5.88	−2.27*
Embarrassment (0 to 3, low to high)	1.02	1.15	1.04	1.20	0.98	1.09	−0.41
Isolation (0 to 4, low to high)	1.36	1.45	1.43	1.49	1.25	1.37	−1.11
Dyadic relationship strain (0 to 4, low to high)	0.43	0.94	0.44	0.99	0.41	0.87	−0.24
Depression (0 to 11, low to high)	2.54	2.38	2.63	2.47	2.40	2.25	−0.85
**Veteran impairment**							
Personal care dependencies (0 to 12, low to high)	1.91	2.49	2.04	2.54	1.71	2.41	−1.43
Cognitive impairment (0 to 28, low to high)	11.24	5.94	11.54	6.28	10.77	5.37	−2.76**
**Background characteristics**							
Age	79.35	7.91	78.72	8.64	80.32	6.54	1.99*
Spouse caregiver	69.1%	–	64.9%	–	75.6%	–	3.23**
Northeast region	41.4%	–	36.1%	–	49.6%	–	2.32*
White	84.8%	–	77.8%	–	95.4%	–	5.06**

*P ≤ .05; **P ≤ .01. PDC, Partners in Dementia Care; St. Dev., standard deviation.

Table 1 also indicates that the PDC and UC groups differed significantly at baseline on four background and context characteristics: veterans’ age; whether veterans had a spouse caregiver versus other relative/friend caregiver or no caregiver; whether veterans were from study sites in the Northeast or Southwest region; and whether veterans were white or a member of a racial minority. These variables were used as covariates in hypothesis testing to statistically control for these differences. In addition, baseline cognitive impairment and unmet need significantly differed between the PDC and UC groups. Cognitive impairment was included and statistically controlled in all regression equations that tested the two hypotheses. Baseline unmet need was included and statistically controlled in analyses that tested whether the PDC and UC groups differed in unmet need after six months.

Table 2 summarized the results of ten regression equations that tested the impact of PDC on veteran outcomes at six and twelve months. To simplify the display of results for the large number of equations, only regression coefficients for the PDC variable and statistically significant product terms representing conditional effects of the intervention are displayed. However, as described in the ‘Analytic Strategy’ section, regression equations included all variables selected for the analysis (that is, the prior-wave version of the dependent variable, two impairment variables and four covariates).

**Table 2:** Summary of ten regression equations that tested the impact of PDC care coordination on six-month and twelve-month veteran outcomes

**Equations**	**Intervention variable**			**Significant product terms with intervention variable**				
**Six-month outcomes**	**B**	**Beta**	***P***		**B**	**Beta**	***P***	**R**^**2a**^
1. Unmet need (number = 223)	-.63	-.05	.44	Intervention x Personal care dependencies	-.52	-.15	.08	.36
				Intervention x Cognitive impairment	-.28	-.22	.02	
2. Embarrassment (number = 255)	-.24	-.11	.08	—				.24
3. Isolation (number = 255)	-.25	-.09	.15	—				.21
4. Relationship strain (number = 232)	.06	.03	.63	Intervention x Personal care dependencies	-.09	-.18	.05	.31
5. Depression (number = 262)	-.20	-.04	.48	Intervention x Cognitive impairment	-.10	-.19	.03	.35
**Twelve-month outcomes**								
6. Unmet need (number = 194)	−1.32	-.12	.12	Intervention x Personal care dependencies	-.96	-.30	<.01	.31
7. Embarrassment (number = 185)	.73	.33	.02	Intervention x Cognitive impairment	-.05	-.39	.02	.27
8. Isolation (number = 185)	-.07	-.02	.76	—				.21
9. Relationship strain (number = 169)	.14	.07	.32	—				.36
10. Depression (number = 187)	.06	.01	.84	—				.50

^a^All are significant at ≤ .01. PDC, Partners in Dementia Care.

Regression equations for six-month outcomes in Table 2 show that veterans receiving PDC compared with those in the UC group had significantly lower levels of four of five outcomes, which is consistent with the study hypotheses. For three outcomes (that is, unmet need, relationship strain and depression), these were significant conditional effects that pertained to more impaired veterans (hypothesis 2). Specifically, among veterans with more personal-care dependencies, six-month unmet need was less for those receiving PDC than for those receiving UC (B = −.52; *P* = .08). Among those more cognitively impaired, six-month unmet need also was less for veterans receiving PDC than for those receiving UC (B = −.28; *P* = .02). For six-month relationship strain, the significant conditional effect was for those with more personal-care dependencies (B = −.09; *P* = .05); veterans receiving PDC had lower levels of this adverse outcome than those receiving UC. For six-month depression, the significant conditional effect was for the more cognitively impaired, with veterans receiving PDC having fewer symptoms of depression than those receiving UC (B = −.10; *P* = .03). The other significant six-month effect of PDC was a main effect (hypothesis 1) for embarrassment about memory problems. Veterans receiving PDC reported lower levels of six-month embarrassment than veterans receiving UC (B = −.24; *P* = .08).

The second part of Table 2 displays results for twelve-month outcomes. There were fewer significant differences between groups after twelve months compared with after six months. Two of the five equations had significant effects. The equation for twelve-month unmet need had a significant conditional effect that was consistent with hypothesis 2. Among those with more personal-care dependencies, veterans receiving PDC compared with UC had fewer unmet needs (B = −.96; *P* < .01).

The other equation with significant differences was for twelve-month embarrassment about memory problems. There were two significant regression coefficients; one was a main effect that was opposite hypothesis 1 (B = .73; *P* = .02). The other was a conditional effect that was consistent with hypothesis 2 (B = −.05; *P* = .02). Interpreted together, these two significant effects suggest that, among less cognitively impaired veterans (that is, average or below-average levels of cognitive impairment in this sample), the PDC group had significantly higher scores than the UC group for twelve-month embarrassment. However, among veterans who were more cognitively impaired (above average levels in this sample), scores for twelve-month embarrassment were similar between the PDC and UC groups. For example, a separate regression analysis of only veterans with higher-than-average cognitive impairment showed no significant group difference between PDC and UC groups for twelve-month embarrassment.

Table 3 further illustrates the statistically significant differences between the PDC and UC groups found in regression analyses. The ‘% change’ column in the Table is particularly informative, since it is not affected by differences in the scoring ranges of the various outcomes. With the exception of results for twelve-month embarrassment, all %-change values in Table 3 for the PDC group are negative (for example, veteran depression = −30.1%), indicating a reduction in adverse outcomes from baseline to six months or from six to twelve months. For the UC group, some %-change values are positive (for example, veteran depression = +50.0%), meaning an increase in adverse outcomes. For other outcomes, %-change values for the UC group are negative but considerably smaller in magnitude than for the PDC group (for example, unmet need – high baseline personal-care dependencies: UC group = −4.6% versus PDC group = −22.2%), meaning less of a decrease in adverse outcomes. Overall, Table 3 illustrates that many different outcomes improved for veterans receiving PDC when compared with veterans receiving UC.

**Table 3:** Illustration of statistically significant differences between PDC and UC groups

**Six-month outcomes with significant change**	**Baseline mean**	**Six-month mean**	**Mean change**	**% Change**
Unmet need–high baseline personal care dependencies				
• UC group	8.7	8.3	−0.4	−4.6%
• PDC group	8.1	6.3	−1.8	−22.2%
Unmet need–high baseline cognitive impairment				
• UC group	5.1	4.7	−0.4	−7.8%
• PDC group	5.9	3.3	−2.6	−44.1%
Embarrassment–all veterans				
• UC group	0.9	0.9	0	0%
• PDC group	1.0	0.8	−0.2	−20.0%
Relationship strain–high baseline personal care dependencies				
• UC group	0.4	0.3	−0.1	−25.0%
• PDC group	0.5	0.1	−0.4	−80.0%
Depression–high baseline cognitive impairment				
• UC group	1.6	2.4	0.8	+50.0%
• PDC group	2.6	1.8	−0.8	−30.1%
**Twelve-month outcomes with significant change**	**Six-month mean**	**Twelve-month mean**	**Mean change**	**% Change**
Unmet need–high six-month personal care dependencies				
• UC group	9.5	9.8	0.3	+3.2%
• PDC group	9.1	3.2	−5.9	−64.8%
Embarrassment–low six-month T2 cognitive impairment				
• UC group	0.9	0.8	−0.1	−11.0%
• PDC group	0.8	0.9	0.1	+12.5%
Embarrassment–high six-month T2 cognitive impairment				
• UC group	1.0	0.9	−0.1	−10.0%
• PDC group	0.8	0.7	−0.1	−12.5%

PDC, Partners in Dementia Care; UC, usual care.

## Discussion

As a study of effectiveness, rather than the more commonly tested efficacy, this research extended the evidence-base for PDC, as well as for BRI Care Consultation (its parent intervention) by: 1) using a larger, more diverse sample than prior studies; and 2) delivering the program in a manner similar to the way it would be implemented if it were part of usual care.

PDC was associated with significantly less adverse outcomes, particularly for more impaired veterans. Improvements in all but one outcome were conditional effects (hypothesis 2) pertaining to veterans who were more cognitively impaired or had more difficulties with personal care. There was one outcome that significantly improved for all veterans receiving PDC (hypothesis 1). Additionally, most beneficial effects of PDC were evident from baseline to six months, with fewer outcomes improving from months six to twelve. However, beneficial effects at six months were maintained throughout the twelve-month study period for all but one outcome (that is, twelve-month embarrassment about memory problems).

Beneficial program effects after six months were evident in reduced relationship strain, depression and unmet need for more impaired veterans, and reduced embarrassment about memory problems for all veterans. Between months six and twelve, there were further reductions in unmet need for more impaired veterans. The one unexpected finding contrary to hypotheses was that embarrassment about memory problems increased for the PDC group between months six and twelve among less cognitively impaired veterans. Embarrassment did not differ between the groups from six to twelve months among more cognitively impaired veterans.

Further research is needed to understand the unexpected findings for embarrassment at twelve months. One possibility is that embarrassment increased for those with mild cognitive impairment because PDC brought attention to symptoms previously normalized, denied or downplayed. PDC initially may have reduced feelings of embarrassment by improving understandings of the diagnosis and symptoms. But, in the longer-term, it may have increased the focus on dementia, which, in turn, may have negatively affected veterans’ feelings about themselves.

This investigation tested the effectiveness of PDC by examining improvements in psychosocial outcomes representing veterans’ subjective perceptions of the consequences of their dementia. Outcomes based on information provided directly by persons with dementia contrast with the more common approach of relying exclusively on proxy reports to estimate their perceptions and experiences [19,20,36]. This measurement approach responds to calls from a growing number of researchers, clinicians, persons with dementia and family caregivers for more studies representing the perspective of persons with dementia, particularly when the disease is mild or moderate [23,37].

Because PDC targeted both persons with dementia and their caregivers, assessing outcomes from both was a logical extension of the program. Asking veterans, along with caregivers, for their opinions also honored the autonomy and dignity of the former and recognized them as active participants in their own care and care-related decisions. At the same time, only a subsample of all enrolled veterans with dementia was able to communicate their perceptions using a structured research data-collection tool, with more severely impaired veterans not represented by the findings.

There were two notable limitations to the study. First, use of matched comparison sites, rather than within-site randomization, made it less certain that the intervention and comparison groups were equivalent at baseline, although observed baseline differences were statistically controlled. Matched sites were used because it facilitated sustainable implementation procedures at PDC sites for subject recruitment and integration of PDC with other services offered by partnering organizations. Second, this investigation did not test whether outcomes differed among veterans within the PDC group, depending on the amounts and types of assistance provided by PDC. Although PDC has a standardized protocol specifying a required minimum exposure to the intervention, the content and number of action steps beyond the minimum varied by individuals and were tailored to veterans’ preferences and needs.

## Conclusions

Positive results from this research suggest that PDC is a promising new model of care coordination. It is being considered for broader implementation by the VA, Alzheimer’s Association, other health systems and community agencies. PDC is consistent with the goals and priorities of a number of national policy and research initiatives charged with developing improved ways of linking healthcare and community services. For example, the National Plan to Address Alzheimer’s Disease [5] has the coordination of health- and community-based care as a key goal [5]. The recent proposed amendment to the Older Americans Act calls for care coordination to link healthcare services and community services [38]. Moreover, positive outcomes achieved by this telephone and computer intervention make PDC a viable approach for assisting hard-to-reach rural populations, another priority of the VA [39].

Large-scale implementation of PDC outside of a research study will require more information on the cost, financing, reimbursement, marketing strategies and ability to integrate it with existing services and information systems [40]. Many of these issues are being examined in a replication study of PDC being conducted in Ohio, with support by the Administration for Community Living (grant 90DS0001). This initiative is being implemented by the Ohio Department of Aging, Benjamin Rose Institute on Aging, Louis Stokes Cleveland Veterans Administration Medical Center, the Western Reserve Area Agency on Aging and the Greater East Ohio Area Alzheimer’s Association Chapter.

## Abbreviations

BRI: Benjamin Rose Institute; CCIS: Care Coordination Information System; PDC: Partners in Dementia Care; UC: usual care; VA: Veterans Administration; VISN: Veterans Integrated Service Networks.

## Competing interests

Authors currently and formerly employed by the non-profit Benjamin Rose Institute on Aging (David Bass, Catherine McCarthy and Wendy Looman) led the research team that developed the BRI Consultation Program, which provided a foundation for Partners in Dementia Care. BRI holds the copyright to and currently licenses and trains organizations to deliver BRI Care Consultation.There are no additional competing interests.

## Authors’ contributions

DMB and MEK: conception and design; acquisition, analysis and interpretation of data; drafting and revising the manuscript; final approval of the version to be published; and agree to be accountable for all aspects of the work; KSJ, ALS, NLW, and CAM: conception and design, acquisition, analysis and interpretation of data; drafting and revising the manuscript; and final approval of the version to be published; ROM: conception and design; analysis and interpretation of data, drafting and revising the manuscript and final approval of the version to be published; KM: analysis and interpretation of data, drafting and revising the manuscript and final approval of the version to be published; RR, JAM, GLO, EA, RE, PP, and TAT: acquisition of data, drafting and revising the manuscript and final approval of the version to be published; WJL: acquisition, analysis and interpretation of data; drafting and revising the manuscript and final approval of the version to be published. All authors read and approved the final manuscript.

## References

[B1] Office of Assistant Deputy Under-Secretary for Health Projections of the Prevalence and Incidence of Dementias Including Alzheimer's Disease for the Total, Enrolled, and Patient Veteran Populations Age 65 and Over 2004 Washington, DC: Department of Veterans Affairs

[B2] Sheets CJ Mahoney-Gleason H Caregiver support in the Veterans Health Administration: caring for those who care Generations 2010 6 92 98

[B3] US Congress Senate. 111th Congress, 2nd Session: Caregivers and Veterans Omnibus Health Services Act of 2010 2010 Washington, DC: GPO

[B4] Cooley SG Asthana S Dementia care for veterans: enhancing comprehensive, coordinated services Generations 2010 6 57 63

[B5] US Department of Health and Human Services The national plan to address Alzheimer’s disease Available at: http://alzheimers.gov/pdf/NationalPlantoAddressAlzheimersDisease.pdf

[B6] Bass DM Clark PA Looman WJ McCarthy CA Eckert S The Cleveland Alzheimer's managed care demonstration: outcomes after 12 months of implementation Gerontologist 2002 6 73 85 10.1093/geront/43.1.73 12604748

[B7] Rosalynn Carter Institute for Caregiving Care consultation telephone-based empowerment intervention Care Consultation Telephone-based Empowerment Intervention. Caregiver Intervention Database 2009 Available at: http://www.rosalynncarter.org/caregiver_intervention_database/dimentia/care_consultation_telephone-based_empowerment_intervention/

[B8] US Administration on Aging (2009) Alzheimer’s Disease Supportive Services Program Evidence-based cooperative agreements to better serve people with Alzheimer’s disease and related disorders OMB Approval No. 0985–0018

[B9] Eloniemi-Sulkava U Saarenheimo M Laakkonen ML Pietilä M Savikko N Kautiainen H Tilvis RS Pitkälä KH Family care as collaboration: effectiveness of a multicomponent support program for elderly couples with dementia. Randomized controlled intervention study J Am Geriatr Soc 2009 6 2200 2208 10.1111/j.1532-5415.2009.02564.x 20121986

[B10] Maslow K Selstad J Chronic care networks for Alzheimer’s disease: approaches for involving and supporting family caregivers in an innovative model of dementia care Alzheimers Care Q 2001 6 33 46

[B11] Garlo K O’Leary JR Van Ness PH Fried TR Burden in caregivers of older adults with advance illness J Am Geriatr Soc 2010 6 2315 2322 10.1111/j.1532-5415.2010.03177.x 21087225 PMC3058825

[B12] Shay K Burris JF Setting the stage for a new strategic plan for geriatrics and extended care in the Veterans Health Administration: summary of the 2008 VA State of the Art Conference, “The changing faces of geriatrics and extended care: meeting the needs of veterans in the next decade” J Am Geriatr Soc 2008 6 2330 2339 10.1111/j.1532-5415.2008.02079.x 19093933

[B13] Bernabei R Landi F Gambassi G Sqadan A Zuccala G Mor V Rubenstein LZ Carbonin P Randomised trial of impact of model of integrated care and case management for older people living in the community BMJ 1998 6 1348 1351 10.1136/bmj.316.7141.1348 9563983 PMC28532

[B14] Czaja SJ Gitlin LN Schulz R Zhang S Burgio LD Stevens AB Nichols LO Gallagher-Thompson D Development of the risk appraisal measure (RAM): a brief screen to identify risk areas and guide interventions for dementia caregivers J Am Geriatr Soc 2009 6 1064 1072 10.1111/j.1532-5415.2009.02260.x 19453305 PMC2722069

[B15] Hepburn KW Tornatore J Center B Ostwald SW Dementia family caregiver training: affecting beliefs about caregiving and caregiver outcomes J Am Geriatr Soc 2001 6 450 457 10.1046/j.1532-5415.2001.49090.x 11347790

[B16] Sink KM Covinsky KE Barnes DE Newcomer RJ Yaffe K Caregiver characteristics are associated with neuropsychiatric symptoms of dementia J Am Geriatr Soc 2006 6 796 803 10.1111/j.1532-5415.2006.00697.x 16696746

[B17] Coon DW Williams MP Moore RJ Edgerly ES Steinbach CM Roth SP Phillips CL Nguyen H Dowling GA Dunning EA Feigenbaum LZ The Northern California Chronic Care Network for Dementia J Am Geriatr Soc 2004 6 150 156 10.1111/j.1532-5415.2004.52026.x 14687331

[B18] Maslow K Translating innovation to impact: evidence-based interventions to support people with Alzheimer’s disease and their caregivers at home and in the community. Administration on aging and alliance for aging research 2012 Available at http://www.nhqualitycampaign.org/files/Maslow_evidencebased%20review%20community%20interventions.pdf. Accessed on 10 October 10, 2013

[B19] Schulz R Cook TB Beach SR Lingler JH Martire LM Monin JK Czaja SJ Magnitude and causes of bias among family caregivers rating Alzheimer disease patients Am J Geriatr Psychiatry 2013 6 14 25 10.1016/j.jagp.2012.10.002 23290199 PMC3330137

[B20] Brod M Steward AL Sands L Walton P Conceptualization and measurement of quality of life in dementia: the Dementia Quality of Life Instrument (DDQoL) Gerontologist 1999 6 25 35 10.1093/geront/39.1.25 10028768

[B21] Pearlin LI Mullan JT Semple SJ Skaff MM Caregiving and the stress process: an overview of concepts and their measures Gerontologist 1990 6 583 594 10.1093/geront/30.5.583 2276631

[B22] Anehensel CS Pearlin LI Mullan JT Zarit SH Whitlatch CJ Profiles of Caregiving: The Unexpected Career 1995 New York: Academic

[B23] Judge KS Menne HL Whitlatch CJ Stress process model for individuals with dementia Gerontologist 2010 6 294 302 10.1093/geront/gnp162 20022935 PMC2867497

[B24] Lin N Lin N, Dean A, Ensel W Modeling the effects of social support Social Support, Life Events, and Depression 1986 Orlando, FL: Academic 173 209

[B25] Bass DM McClendon MJ Brennan PF McCarthy C The buffering effect of a computer support network on caregiver strain J Aging Health 1998 6 20 43 10.1177/089826439801000102 10182416

[B26] Katzman R Brown T Fuld P Peck A Schechter R Schimmel H Validation of a short Orientation-Memory-Concentration test of cognitive impairment Am J Psychiatry 1983 6 734 739 6846631 10.1176/ajp.140.6.734

[B27] US Department of Health and Human Services Early Identification of Alzheimer’s Disease and Related Dementias 1996 Agency for Health Care Policy and Research Publication No. 97–070324506061

[B28] Judge KS Bass DM Snow AL Wilson NL Morgan R Looman WJ McCarthy C Kunik ME Partners in Dementia Care: a care coordination intervention for individuals with dementia and their family caregivers Gerontologist 2011 6 261 272 10.1093/geront/gnq097 21242317

[B29] Krestar ML Loman W Powers S Dawson S Judge KS Including individuals with memory impairment in the research process: the importance of scales and response categories used in surveys J Empir Res Hum Res Ethics 2012 6 70 79 10.1525/jer.2012.7.2.70 22565585

[B30] Clark PA Bass DM Looman WJ McCarthy CA Eckert S Outcomes for patients with dementia from the Cleveland Alzheimer’s managed care demonstration J Aging Ment Health 2004 6 40 51 10.1080/13607860310001613329 14690867

[B31] Bass DM McClendon MJ Deimling GT Mukherjee S The influence of diagnosed mental impairment on family caregiver strain J Gerontol 1994 6 S146 S155 10.1093/geronj/49.3.S146 8169349

[B32] Kohout FJ Berkman LF Evans DA Cornoni-Huntley J Two shorter forms of the CES-D (Center for Epidemiological Studies Depression) depression symptoms index J Aging Health 1993 6 179 193 10.1177/089826439300500202 10125443

[B33] Lawton MP Brody EM Assessment of older people: self-maintaining and instrumental activities of daily living Gerontologist 1969 6 179 186 10.1093/geront/9.3_Part_1.179 5349366

[B34] McClendon M Multiple Regression and Causal Analysis 1994 Waveland: Prospect Heights, IL

[B35] Cohen J Cohen P Applied Multiple Regression/Correlation Analysis for the Behavioral Sciences 1975 Mahwah, NJ: Lawrence Erlbaum

[B36] Dawson NT Powers SM Krestar M Yarry SJ Judge KS Predictors of self-reported psychosocial outcomes in individuals with dementia Gerontologist 2013 6 748 757 10.1093/geront/gns137 23107792 PMC3771672

[B37] Reed P Bluethmann S Voices of Alzheimer’s Disease: A Summary Report on the Nationwide Town Hall Meetings for People with Early Stage Dementia 2008 Chicago: Alzheimer’s Association

[B38] S. 3465–112th Congress Care Coordination for Older Americans Act of 2012. (August 30, 2012) GovTrack.us (database of federal legislation) 2012 Available at http://www.govtrack.us/congress/bills/112/s3465

[B39] Strategic plan refresh. FY 2011–2015 Washington, DC: US Department of Veterans Affairs Available at http://www.va.gov/VA_2011-2015_Strategic_Plan_Refresh_wv.pdf. Accessed October 10, 2013

[B40] Bass DM Judge KS Challenges implementing evidence-based programs Generations 2010 6 51 58

